# Successful Complete Response of Tumor Thrombus after Combined with Chemotherapy and Irradiation for Ewing Sarcoma

**DOI:** 10.1155/2018/5238512

**Published:** 2018-01-21

**Authors:** Yusuke Minami, Seiichi Matsumoto, Keisuke Ae, Taisuke Tanizawa, Keiko Hayakawa, Yuki Funauchi, Sakae Okumura, Yutaka Takazawa

**Affiliations:** ^1^Department of Orthopedic Surgery, The Cancer Institute Hospital of the Japanese Foundation for Cancer Research, Tokyo, Japan; ^2^Department of Thoracic Surgery, The Cancer Institute Hospital of the Japanese Foundation for Cancer Research, Tokyo, Japan; ^3^Department of Pathology, The Cancer Institute Hospital of the Japanese Foundation for Cancer Research, Tokyo, Japan

## Abstract

Pelvic Ewing sarcoma is associated with a worse prognosis. Thromboembolic events are relatively common in pediatric patients with cancers including sarcomas. We have presented a case of Ewing sarcoma arising from the left iliac bone with tumor thrombus of inferior vena cava (IVC) which was obtained complete response by both chemotherapy and irradiation. Magnetic resonance imaging (MRI) scan demonstrated that the tumor arising from the left iliac bone extended into the left side of sacral bone, suggesting the difficulty of surgical resection. Computed tomography (CT) revealed the existence of the tumor thrombus of IVC. We performed irradiation (31.2 Gy) and chemotherapy (combination of VCR, Act-D, IFM, and ADR). The tumor was controlled successfully, and the tumor thrombus of IVC has completely vanished. Four years after the treatment, coin lesion in the left upper lung appeared. Suspected of metastasis, segmental resection of the left upper lung was performed. Fourteen years after the surgery, the patient has been remained free of recurrence. It is clinically significant for surgeons to treat pelvic Ewing sarcoma with tumor thrombus.

## 1. Introduction

Ewing sarcoma family of tumors (ESFTs) are rare, but high-grade malignant tumors of unclear etiology which mainly occurs in childhood [1]. Particularly, pelvic Ewing sarcoma commonly resulted in a poor prognosis [2]. Cancer and thromboembolic events are strongly associated. Venous thromboembolic events are often occurred in pediatric patients with sarcomas [3]. A few papers have been reported so far in sarcomas with tumor thrombus: osteosarcomas [4], [5], chondrosarcomas [6], clear cell sarcomas [7–9], leiomyosarcomas [10–12], rhabdomyosarcomas [13], and liposarcomas [14]. As far as we investigated, no case report of tumor thrombus with Ewing sarcomas has been reported.

## 2. Case Presentation

**Figure 1:**
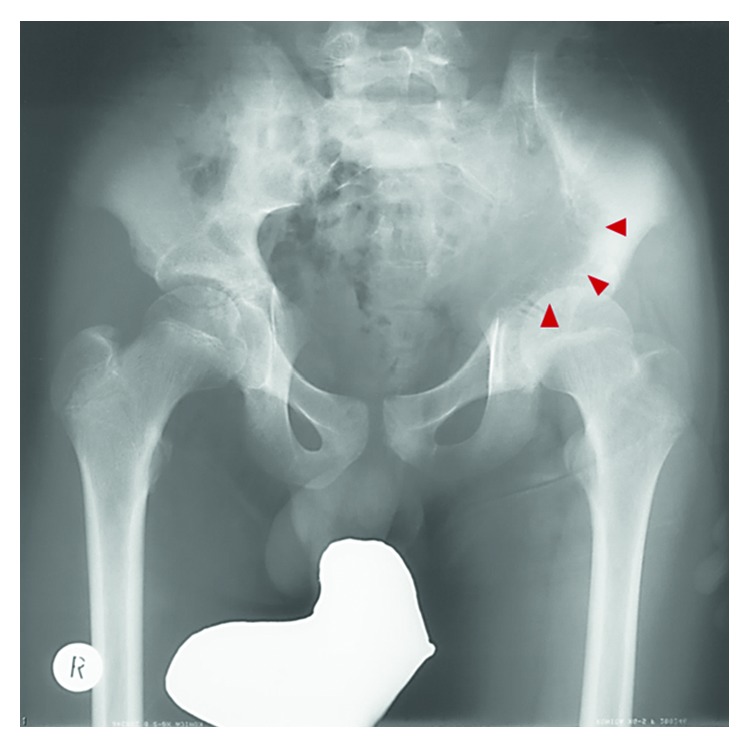
Plain radiograph showing osteolytic changes at the medial side of the left iliac bone.

**Figure 2:**
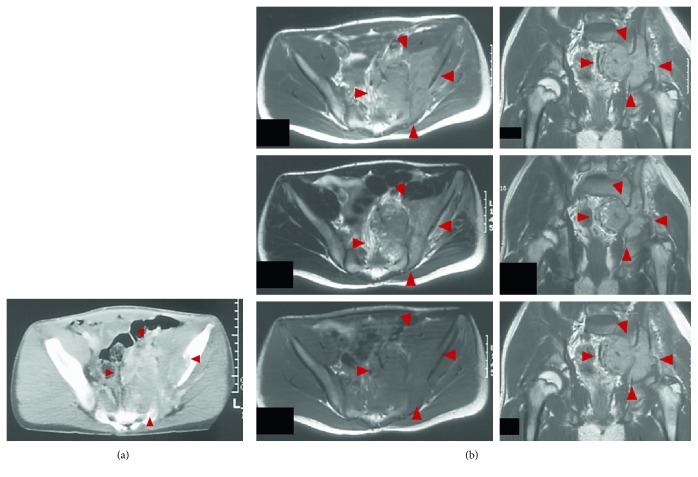
(a) 7.2 × 9.5 × 3.0 cm mass arising from the left iliac bone and extended into the sacral bone. (b) The large mass showing low intensity on T1-weighted images and high intensity on T2-weighted images and sparsely enhanced by contrast agent.

**Figure 3:**
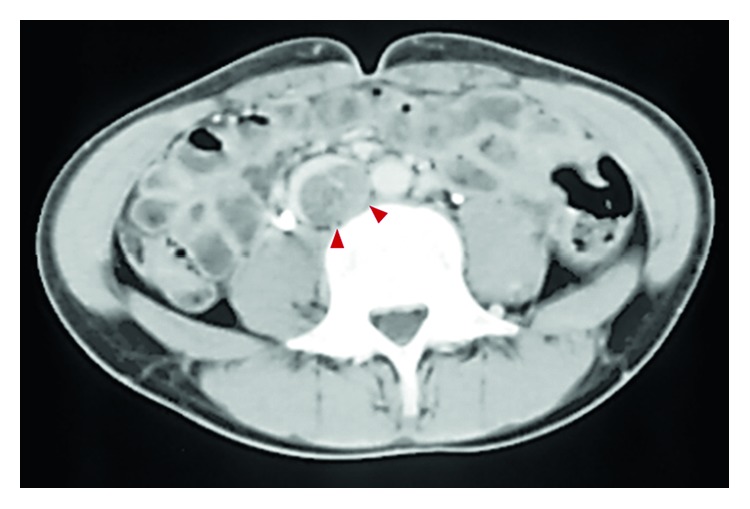
A contrast-enhanced CT suggesting the existence of the tumor thrombus of IVC.

**Figure 4:**
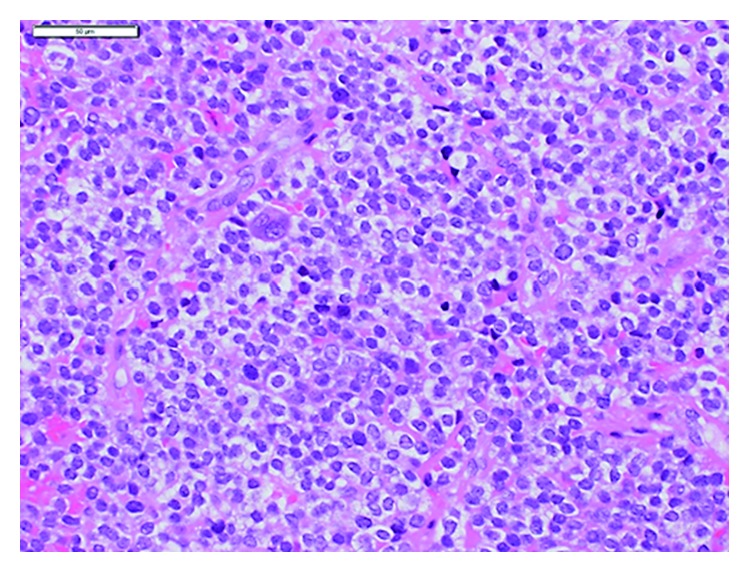
The tumor cells are small and round shaped and have abundant glycogen.

**Figure 5:**
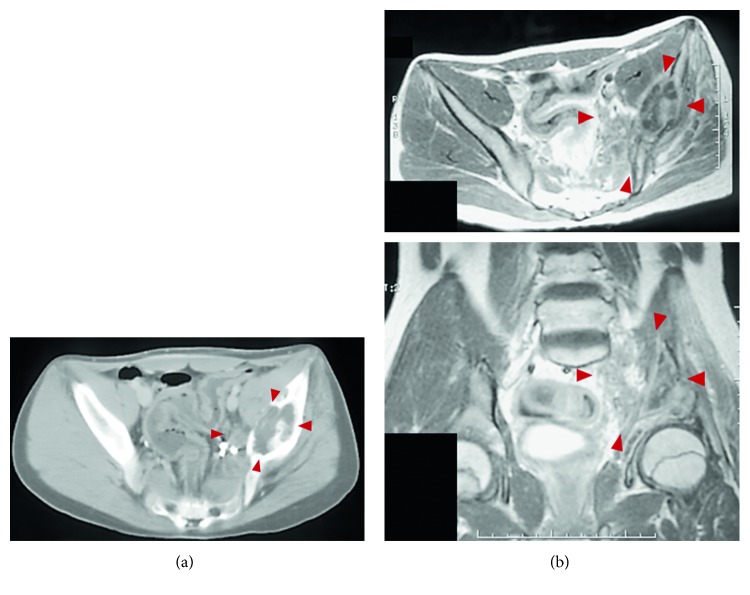
(a, b) The tumor volume was reduced successfully and judged as partial response (PR).

**Figure 6:**
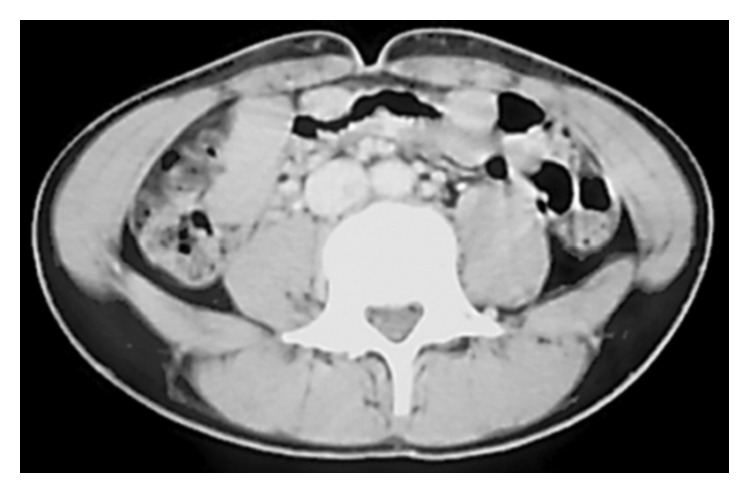
The tumor thrombus of IVC has completely vanished after irradiation and a 1 cycle of chemotherapy.

**Figure 7:**
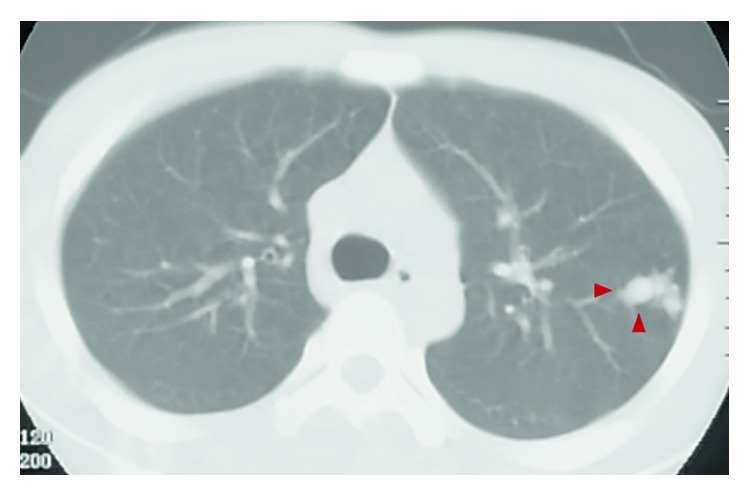
Four years after the treatment, coin lesion in the left upper lung appeared.

**Figure 8:**
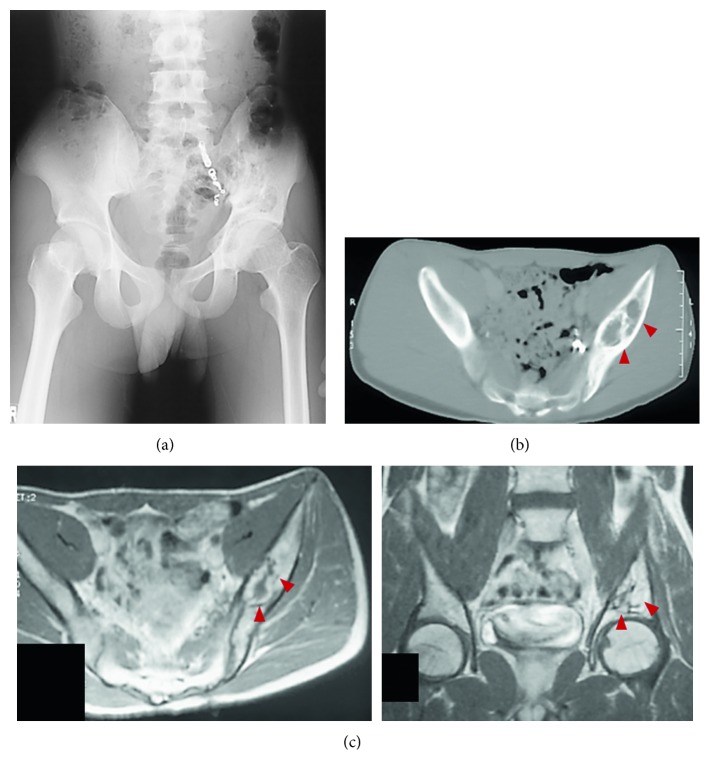
(a–c) 14 years after the surgery, the patient has remained with no evidence of recurrence.

A 14-year-old male with continuous left low back pain, suspected of malignancy, referred to our hospital. He had been aware of left low back pain for the last 3 months. A clinical examination revealed spontaneous pain of his left low back and the paresthesia of left S1, 2 lesion. Plain radiograph of the pelvic bone showed osteolytic changes of the left iliac bone (Figure 1). CT and MRI demonstrated that a 7.2 × 9.5 × 3.0 cm tumor arising from the left iliac bone extended into the left side of sacral bone (Figures 2(a) and 2(b)). The large mass showed low intensity on T1-weighted images and high intensity on T2-weighted images and was enhanced by contrast agent (Figure 2(b)). A contrast-enhanced CT suggested the existence of the tumor thrombus of IVC (Figure 3). Open biopsy of the tumor was performed. Histopathologically, the tumor cells were small and round shaped and have abundant glycogen (Figure 4). Immunostaining analysis showed MIC2 positive and NSE positive. The histopathological diagnosis was Ewing sarcoma. Before treatment, insertion of permanent IVC filter was performed to prevent fatal pulmonary embolism [15]. We performed irradiation (total 31.2 Gy) and total 4 cycles of chemotherapy (combination of VCR, Act-D, IFM, and ADR) because it was considered to be difficult to resect the mass surgically. After irradiation and a 1 cycle of chemotherapy, the tumor volume was reduced successfully and radiologically evaluated as partial response (PR) by both CT and MRI (Figures 5(a) and 5(b)). Moreover, the tumor thrombus of IVC has completely vanished (Figure 6). In addition, we performed 3 cycles of chemotherapy. 4 years after the treatment, coin lesion of the left upper lung appeared (Figure 7). Suspected of lung metastasis, segmental excision of the left upper lung was performed. The histopathological diagnosis was equally Ewing sarcoma. 14 years after the surgery, the patient has been remained free of any evidence of recurrence and tumor thrombus (Figures 8(a)–8(c)).

## 3. Discussion

The combination of surgery, chemotherapy, and irradiation for pelvic Ewing sarcoma has only resulted in about 40% five-year survival rate [16]. We have presented a case of Ewing sarcoma arising from the left iliac bone which caused tumor thrombus of inferior vena cava (IVC). The tumor was obtained complete response by both chemotherapy and irradiation. The tumor thrombus of IVC has vanished without any anticoagulant therapy, which indicates that the content of IVC was not a venous thrombus but the tumor thrombus. We did not perform surgical procedures except for segmental excision of the left upper lung for lung metastasis. Previous papers showed that there was no significant difference in outcomes of patients with pelvic Ewing sarcoma treated with surgery and/or irradiation, although surgical resection was associated with superior outcomes for osteosarcoma and chondrosarcoma [17]. In this case, if we performed the curative surgery, both left hip amputation and tumor evacuation of IVC should be required. The surgical procedure itself might be very difficult and risky for the patient. Moreover, small round cell sarcomas such as Ewing sarcomas are known to be relatively sensitive to chemotherapy and irradiation [18]. On the other hand, spindle cell sarcomas require the curative surgery.

It is clinically very important that the pelvic Ewing sarcoma with the tumor thrombus of IVC could be controlled successfully by irradiation and chemotherapy without any surgical procedure. Our case report should be taken into consideration for surgeons to decide the treatment of pelvic Ewing sarcomas with tumor thrombus.
